# Hereditary renal adysplasia, pulmonary hypoplasia and Mayer-Rokitansky-Küster-Hauser (MRKH) syndrome: a case report

**DOI:** 10.1186/1750-1172-5-6

**Published:** 2010-04-14

**Authors:** Pedro Acién, Francisco Galán, Irene Manchón, Eva Ruiz, Maribel Acién, Luis A Alcaraz

**Affiliations:** 1 Service of Obstetrics and Gynecology, University Hospital of San Juan; Department of Gynecology, "Miguel Hernández" University, Campus of San Juan, Alicante, Spain; 2 Centro de Genética Humana. Alicante, Spain; 3 Bioarray S.L., Alicante, Spain

## Abstract

**Background:**

Hereditary renal adysplasia is an autosomal dominant trait with incomplete penetrance and variable expression that is usually associated with malformative combinations (including Müllerian anomalies) affecting different mesodermal organs such as the heart, lung, and urogenital system.

**Case report:**

A case showing pulmonary hypoplasia, hip dysplasia, hereditary renal adysplasia, and Mayer-Rokitansky-Kuster-Hauser syndrome in adulthood is reported here. The i.v. pyelography showed right renal agenesis with a normal left kidney and ureter. Ultrasound and Magnetic Resonance Imaging also showed right renal agenesis with multicystic embryonary remnants in the right hemipelvis probably corresponding to a dysgenetic kidney. An uretrocystoscopy showed absence of ectopic ureter and of the right hemitrigone. She was scheduled for a diagnostic laparoscopy and creation of a neovagina according to the McIndoe technique with a prosthesis and skin graft. Laparoscopy confirmed the absence of the uterus. On both sides, an elongated, solid, rudimentary uterine horn could be observed. Both ovaries were also elongated, located high in both abdominal flanks and somewhat dysgenetics. A conventional cytogenetic study revealed a normal female karyotype 46, XX at a level of 550 GTG bands. A CGH analysis was performed using a 244K oligoarray CGH detecting 11 copy number variants described as normal variants in the databases. The 17q12 and 22q11.21 microdeletions described in other MRKH patients were not present in this case. Four years after operation her evolution is normal, without symptoms and the neovagina is adequately functional. The geneticists have studied her family history and the pedigree of the family is shown.

**Conclusions:**

We suggest that primary damage to the mesoderm (paraaxil, intermediate, and lateral) caused by mutations in a yet unidentified gene is responsible for: 1) skeletal dysplasia, 2) inappropriate interactions between the bronchial mesoderm and endodermal lung bud as well as between the blastema metanephric and ureteric bud, and eventually 3) Müllerian anomalies (peritoneal mesothelium) at the same level. These anomalies would be transmitted as an autosomal dominant trait with incomplete penetrance and variable expressivity.

## Introduction

According to previous studies [1], unilateral renal agenesis (RA) is embryologically associated with genital and sometimes extragenital malformations. The associated genital malformations are due either to agenesis or hypoplasia of all derivatives of the ipsilateral urogenital ridge (frequently with unicornuate uterus on the opposite side) or to distal mesonephric anomalies [2]. But involution or secondary renal atrophy without uterine malformation is an alternative embryologic sequence resulting in unilateral RA without Müllerian anomalies. Malformative combinations (including Müllerian anomalies) can sometimes affect different organs derived from the mesoderm, such as the heart, lung, and urogenital system [3]. The Mayer-Rokitansky-Küster-Hauser (MRKH) syndrome is a malformation of the female genitals (occurring in one in 4000 female live births) as a results of interrupted embryonic development of the Müllerian ducts [4]. Strübbe et al. [5] divided their MRKH syndrome patients into two groups: typical (isolated form of congenital agenesis of the vagina and uterus), and atypical form, suggesting to call this last type the GRES (genital, renal, ear, skeletal) syndrome. More recently, Oppelt et al. [4] have also classified their 53 cases of MRKH syndrome in three recognized subtypes: typical, atypical and MURCS (Müllerian duct aplasia, renal aplasia, and cervicothoracic somite dysplasia) association. And of the 521 cases included in the revision they do of the literature, 64% were typical, 24% atypical and 12% MURCS. Malformations of the renal system were the most frequent type of accompanying malformation, with 23 different malformations in 19 patients, followed by 19 different skeletal changes in 15 patients of the Oppelt et al's cases. They do not mention cases with pulmonary hypoplasia.

In this communication, we report the case of a patient presenting right hip dysplasia, congenital right pulmonary hypoplasia (PH), and hereditary renal adysplasia with multicystic embryonary remnants in the right hemipelvis (dysgenetic kidney, mesoderm dysplasia) in addition to the Müllerian anomaly known as Rokitansky or MRKH syndrome.

## Case Report

A 17-year-old woman with primary amenorrhea was sent to us with a diagnosis of Rokitansky syndrome. The patient was born via normal delivery at a weight of 2400 g when her mother was 17. She was admitted to the hospital at an age of 8 days due to vomiting and moderate dystrophy; she was then diagnosed with primary right PH. She was later readmitted several times for pulmonary insufficiency. Two months later, a diagnosis of congenital right pulmonary hypoplasia with hypoplasia of the right lung artery was confirmed. In an ultrasound examination performed 7 months later, the right kidney was not observed. From more recent (at an age of 15 years) X-ray images taken of the pelvis in the Emergency Unit, the patient was also diagnosed with right hip dysplasia; this condition was initially defined as old secondary osteonecrosis of the right femoral head.

**Figure 1:**
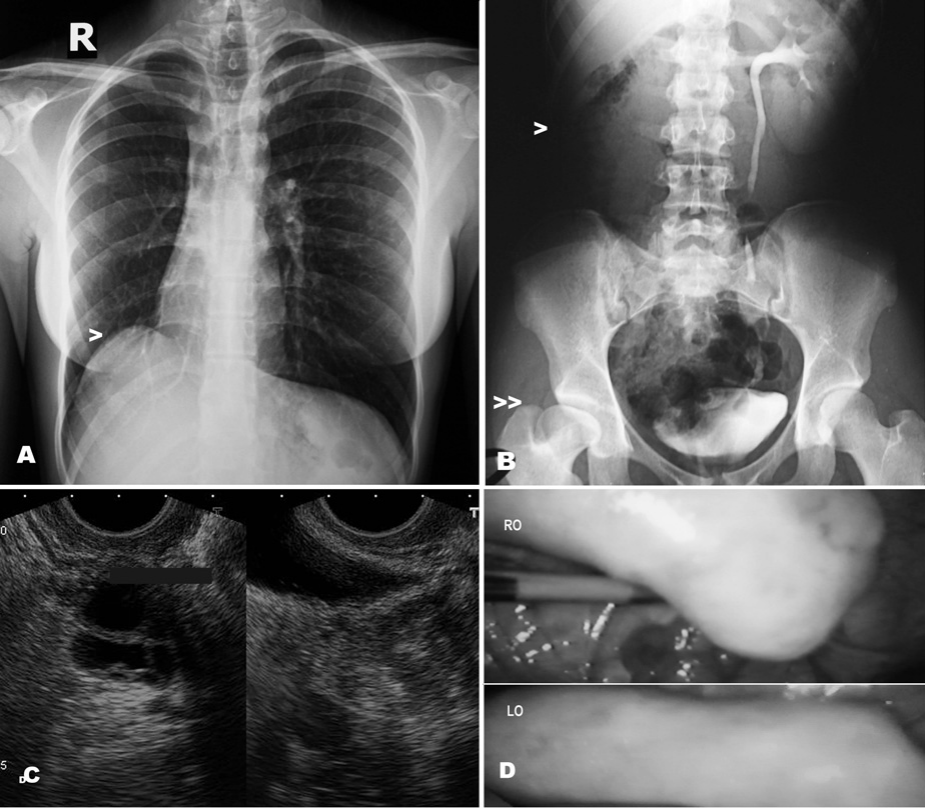
**A. Antero-posterior X-ray of the thorax showing right pulmonary hypoplasia**. Ascent of the diaphragm (>) and displacement of the heart to the right. B. I.V. pyelography showing right renal agenesis (or dysplasia) (>). The right hip dysplasia is also shown (>>). C. Transrectal ultrasound image of the supposed right ureterocele. D. Laparoscopic images of both ovaries (RO, right ovary; LO, left ovary).

**Figure 2:**
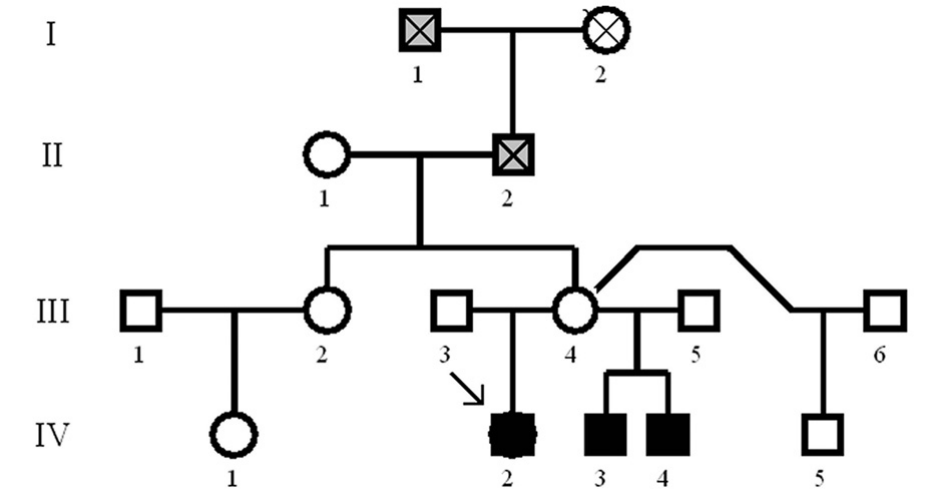
**Pedigree of the family**. The proband's mother (III_4_), who is an obligate carrier, had a normal renal US scan and normal thoracic radiographs. She had three marriages (III_3_, _5_, _6_) and four gestations, including IV_2 _(index case) and IV_3_, _4 _(two boys aged 15 and 5, respectively). In IV_3_, right renal agenesis was diagnosed. We have not observed associated genital anomalies. IV_4 _was prenatally diagnosed at 20 weeks with a dysplastic right kidney. He has associated genital anomalies (absent right testis). Subject IV_5 _has a normal phenotype. The mother and sister of III_4 _have normal US scans and radiographs. III_4 _described lung hypoplasia diagnosed at autopsy in her father (II_2_) and grandfather (I_1_). Grey squares indicate lung hypoplasia reported by subject III_4 _but not documented with a clinical history.

The patient reported three years of genital and mammary development as well as cyclic pelvic pain for 4-5 days every month despite the primary amenorrhea. She weighed 42 kg and was 153 cm tall. The physical examination revealed normal external genital development and normal breasts. There was complete vaginal atresia. In the combined rectal examination, the pelvis was noted to be free. A transrectal ultrasound did not confirm the presence of a uterus. In this ultrasound examination, there was difficulty visualizing the ovaries; on the right side, however, vascular dilatations or multicystic embryonary remnants could be observed. The abdominal ultrasound did not show the presence of a right kidney. General and hormonal analyses were normal (FSH 3.9 mUI/mL, LH 6.2 mUI/mL, PRL 7.9 ng/mL, E2 35 pg/mL, P 0.3 ng/mL), and chest X-rays and i.v. pyelography showed right pulmonary hypoplasia, dysplasia of the right hip, and right renal agenesis with a normal left kidney and ureter (see Figure 1). Later, a conventional cytogenetic study revealed a normal female karyotype 46, XX at a level of 550 GTG bands. A CGH analysis was performed using a 244K oligoarray CGH (Agilent Technologies, Santa Clara, CA, USA), detecting 11 copy number variants (CNV) described as normal variants in the databases. Some CNVs (17q12 and 22q11.21 microdeletions) described in other MRKH patients were not present in this case. The geneticists have been studying her family history and Figure 2 shows the pedigree of the family, compatible with an autosomal dominant pattern of inheritance.

Eight months later, a new transrectal ultrasound was performed. Again, there was no evidence to suggest any functioning rudimentary uterine horn. The right ovary appeared to be normal, and there were the same vascular dilatations or multicystic remnants previously seen on the right side. The left ovary was also difficult to identify properly. The patient, who visited the clinic with her parents, wished operation and to have a neovagina created. She was therefore scheduled for a diagnostic laparoscopy and creation of a neovagina according to the McIndoe technique with a prosthesis and skin graft.

**Figure 3:**
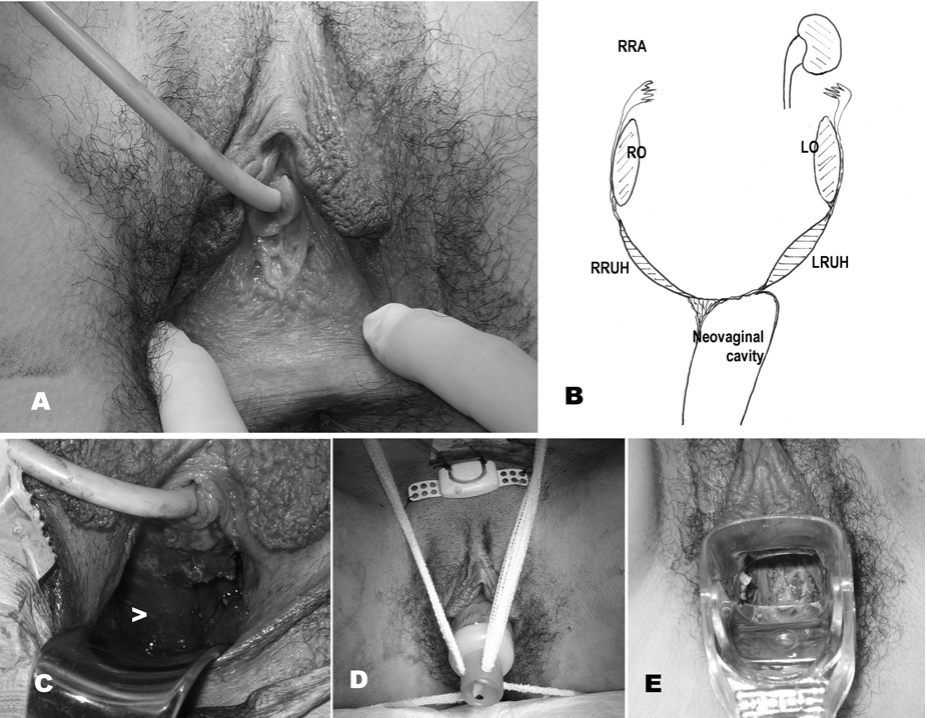
**A. Vulva and vestibule showing the vaginal agenesis**. B. Drawing (diagram) of the genito-urinary anomaly of the patient (RRA, right renal agenesis; RO, right ovary; LO, left ovary; RRUH, right rudimentary uterine horn; LRUH, left rudimentary uterine horn). C. Surgical dissection and formation of the neovaginal cavity (>). D. Prosthesis with skin graft placed in situ. E. Observation of the epidermization of the neovagina 10 days after surgery.

**Figure 4:**
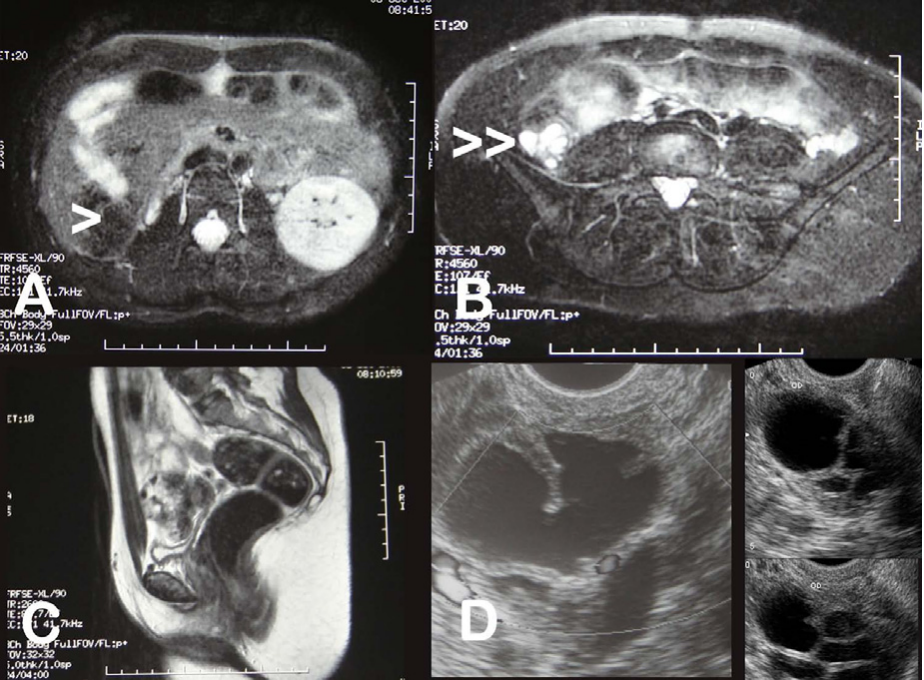
**A and B: Magnetic Resonance Imaging showing the right renal agenesis (>) and the multicystic embryonary remnants in the right hemipelvis (>>)**. C. Medial cut in the axial plane. Absence of the uterus. D, Multicystic images in the right hemipelvis (probably a dysgenetic kidney) by transvaginal ultrasound four years after operation.

Laparoscopy confirmed the absence of uterus. There was a varicocele and/or retroperitoneal multicystic formations on the right side of the small pelvis. On both sides, an elongated, solid, rudimentary uterine horn could be observed. At its superior end, there were ovaries that were elongated, somewhat dysgenetic (more on the right side), and located very high in both abdominal flanks (Figure 1D). Rudimentary tubes could also be observed. Although the uterine rudimentary tract was slightly thicker on the left side, there did not appear to be even minimal endometrial cavitation of the solid uterine rudimentary horn. Sectioning and dissection from the introitum to the inside below the urethra was performed via a transperineal procedure, thus forming a wide vaginal neocavity reaching the Douglas pouch. Interestingly, the dissection of the neovaginal cavity tended more toward the left fundus than the right, as if there were more atresia on the right side of the supposed location of the vagina. A prosthesis with a skin graft taken from the right buttock was applied, with Interceed^® ^placed between the skin and the prosthesis (Figure 3). The drawing of the genital tract of the patient generated in the surgery theater is shown in Figure 2B. After 10 days, the prosthesis was removed and the state of the neovagina was assessed. After replacement of the prosthesis, the patient was discharged. The patient returned for a follow-up visit 6 months after the operation, and her evolution was normal. She then underwent transvaginal ultrasound that did not show the ovaries properly but showed the multicystic embryonary remnants in the right hemipelvis. An uretrocystoscopy showed absence of ectopic ureter and of the right hemitrigone. Likewise, the Magnetic Resonance Imaging (MR) showed right renal agenesis with probable embryonary remnants of a disgenetic kidney in the right hemipelvis with a multicystic morphology (see Figure 4). Those cysts seem to have slightly increased in the transvaginal ultrasound, but four years after operation her evolution is normal, without symptoms and the neovagina is adequately functional.

## Discussion

Renal agenesis and dysplasia (both unilateral and bilateral) are frequently observed in fetuses and newborns, and have been commonly considered by pathologists and pediatricians to be sporadic malformations. However, hereditary renal adysplasia is an autosomal dominant trait with incomplete penetrance and variable expression [6-8]. It can be associated with other heart, lung, or genital anomalies. Its penetrance is between 50% and 90%, and ultrasound study of the kidneys of parents, siblings, and other relatives is recommended in all families with unilateral or bilateral RA individuals [7].

Pulmonary hypoplasia (PH) consists of the presence of variable amounts of pulmonary primordium, bronchial tree, and supporting vasculature. Bilateral PH is a major cause of neonatal mortality and morbidity [9]. Unilateral PH is a defect compatible with an almost normal existence though, and its associated defects govern the prognosis of the patients. Unilateral lung malformation is frequently associated with other singular or complex anomalies (e.g. renal and vascular) [10].

Mirapeix et al [11] noted that the association between bilateral RA and bilateral PH is well known, but that unilateral agenesis of both organs is rare, despite the fact that five cases had been observed in neonatal and infant necropsies. As an unusual association present in adulthood, a case was reported of left RA and left PH in a 46-year-old woman. These concurrent deformities suggested interference with embryologic development, and the morphogenesis of both structures (i.e., the lungs and kidneys) was suggested to have three points in common: 1) they require the induction of the mesoderm on the bronchial and ureteric buds [11,12]; 2) they develop during the same period [13]; 3) for normal lung development, the presence of a pulmonary growth factor produced by the kidney is required [14,15]. However, the relationship of the developing lung and kidney is not completely understood. Renal enlargement has been reported in association with pulmonary hypoplasia in congenital diaphragmatic hernia [16].

As we pointed out in the introduction, unilateral RA (and its associated genital anomalies) generally arises due to either: a) agenesis or hypoplasia of a urogenital ridge or b) distal mesonephric anomalies with early degeneration or absence of the ureteric bud that result in a lack of induction of the metanephric blastema [17,18]. The lung bud appears in the 4^th ^week of fetal life as a median laryngo-tracheal groove in the ventral wall of the foregut [19]; by the 5^th ^week, the lung bud starts branching [13]. This process requires an interaction between the specific bronchial mesoderm and the bud endoderm [12]. Moreover, for normal early lung growth, several investigators [20] have suggested that the presence of the kidney is important.

Mirapeix et al [11] also noted that the simultaneous occurrence of both malformations (RA and PH) might be the result of 1) the simultaneous action of two or more teratogens, 2) the presence of one malformation (RA) that induces the other (PH), or 3) the presence of one teratogen that damages several developmental processes during the 5^th ^week. These mechanisms agree with Källen and Winberg's hypothesis [21] that variable cranio-caudal extension of primary damage in the **mesoderm **could explain the different manifestations of Potter's syndrome as well as associated anomalies.

Müllerian aplasia (Rokitansky or MRKH syndrome) has been defined as an absent or rudimentary uterus and upper vagina in 46, XX females with normal secondary sexual development. As pointed out above, more than 30% of these women have associated defects, with renal and skeletal anomalies occurring most frequently. Although most cases are isolated, familial cases with autosomal dominant transmission, incomplete penetrance, and variable expressivity account for more than 20% of cases. Some candidate genes and chromosome regions (e.g., HOXA7-13, PBX1, EMX2, PAX2, WT1, 10q26, and 6p) have been studied [22,23]; however, clear associations have not yet been established. Recently, an array-based comparative genomic hybridization study detected molecular imbalances, including 22q11.21, 17q12, and Xq21.31 deletions and a 1q21.1 duplication [24]. Other two MRKH patients described recently [25] carry the same 1.5 Mb de novo 17q12 microdeletion, including TCF2 and LHX1 genes. The other 20 MRKH patients in this study, as in our case, do not carry the microdeletion and sequencing of TCF2 and LHX1 genes does not detect pathological mutations. All these data suggest a wide genetic heterogeneity for MRKH syndrome.

Therefore, it is possible that primary damage to the mesoderm (paraaxil: muscle, skeletal; intermediate: urogenital; lateral: lung, heart, and serosal membranes) originates from: 1) inadequate interaction between the bronchial mesoderm and endodermal lung bud; 2) inadequate interaction between the metanephric blastema and ureteric bud (which, rather than pulmonary or renal agenesis, would then cause hypoplasia and dysgenetic kidney); 3) eventual Müllerian anomalies at that same level. It is important to remember that the Müllerian or paramesonephric ducts are formed from longitudinal invaginations of the mesothelium on the lateral face of the urogenital ridge and mesonephros. Such mesothelial tissues (specifically, the serous membranes of the pleura, pericardium, and peritoneum) are derived from the lateral mesoderm [3]. In this case, our patient also presented dysplasia of the right hip (skeletal dysplasia), which likewise suggested damage to the paraaxil mesoderm.

Mirapeix et al. [11] did not describe the genitals of the 46-year-old woman they studied with pulmonary hypoplasia and RA, and the genital tracts in other cases with neonatal death (almost always with family antecedents of RA) were not described either [26,27]. However, Schimke et al. [28] previously reported a family in which three-generation transmission of renal agenesis-dysplasia occurred with no skeletal defects but with uterine anomalies. Pavanello et al. [29] also reported a relationship between Rokitansky syndrome and hereditary renal adysplasia. Likewise, Battin et al. [30] reported a family with unilateral or bilateral RA and Müllerian anomalies. Strubbe et al. [5] studied 100 cases with MRKH syndrome and, as we said before, divided the patients into two groups (i.e., a typical and atypical form) suggesting that this type of MRKH syndrome should be called GRES (genital, renal, ear, skeletal) syndrome. Carranza-Lira et al. [31] also described three patients with MRKH syndrome and a MURCS (Müllerian renal cervical somite) association. Finally, Morcel et al. [32] also described that MRKH may be isolated (type I) but it is more frequently associated with renal, vertebral, and, to a lesser extent, auditory and cardiac defects (MRKH type II or MURCS association). The etiology still remains unclear.

Nonetheless, simultaneous pulmonary hypoplasia, renal agenesis or dysplasia, and MRKH syndrome have not been observed in adulthood; further, our case also presented hip dysplasia. The multicystic embryonary remnants in the right hemipelvis suggest a dysgenetic kidney and secondary involution rather than an actual RA due to agenesis of the urogenital ridge on one side or distal mesonephric anomaly.

## Conclusions

We suggest that primary damage to the mesoderm (paraaxil, intermediate, and lateral) caused by mutations in a yet unidentified gene is responsible for skeletal, pulmonary, renal, and for Müllerian dysplasias present in MRKH syndrome and usually transmitted as an autosomal dominant trait with incomplete penetrance and variable expressivity.

## Consent

Written informed consent was obtained from the patient for publication of this case report and accompanying images. A copy of the written consent is available for review by the Editor-in-Chief of this Journal.

## Competing interests

The authors declare that they have no competing interests.

## Authors' contributions

PA and ER were the gynecologist that operated and followed the patient. PA conceived the study and participated in its design and coordination and wrote the draft of the manuscript. MA participated in the design of the study and reviewed the literature. FG and IM made the genetic studies, studied the family history and carried out the pedigree of the family. LAA performed the oligoarray CGH analysis. All authors have contributed equally to this study. All authors read and approved the final manuscript.

PA had full access to all of the data in the study and takes responsibility for the integrity of the data and the accuracy of the data analysis.
